# Web-Based Skin Cancer Prevention Training for Massage Therapists: Protocol for the Massage Therapists Skin Health Awareness, Referral, and Education Study

**DOI:** 10.2196/13480

**Published:** 2019-05-15

**Authors:** Lois J Loescher, Kelly M Heslin, Laura A Szalacha, Graciela E Silva, Myra L Muramoto

**Affiliations:** 1 College of Nursing University of Arizona Tucson, AZ United States; 2 College of Public Health University of Arizona Tucson, AZ United States; 3 College of Medicine University of South Florida Tampa, FL United States; 4 College of Nursing University of South Florida Tampa, FL United States; 5 College of Medicine University of Arizona Tucson, AZ United States

**Keywords:** skin cancer, primary prevention, secondary prevention, health education, e-learning, massage, web-based learning, massage therapists

## Abstract

**Background:**

Skin cancer, the most common cancer in the United States, is costly and potentially deadly. Its burden can be reduced by early detection and prevention activities. The scope of skin cancer requires going beyond traditional health care providers to promote risk reduction. Partnering with the nonbiomedical workforce, such as massage therapists (MTs), may reach more individuals at risk. MTs see much of their clients’ skin and are amenable to performing skin cancer risk reduction activities during massage appointments.

**Objective:**

The objective of this study is to describe the Massage Therapists Skin Health Awareness, Referral, and Education protocol, presenting an overview of our systematic approach to developing rigorous e-training for MTs to enable them to be partners in skin cancer risk reduction. We also describe procedures for usability and feasibility testing of the training.

**Methods:**

We developed an integrated electronic learning system that includes electronic training (e-training) technology, simulated client interactions, online data collection instruments, and in-person assessment of MTs’ application of their training.

**Results:**

A total of 20 participants nationally scored the e-training as high for usability and satisfaction. We have screened an additional 77 MTs in Arizona for interest and eligibility, and currently have 37 enrolled participants, of whom 32 have completed the Web-based training.

**Conclusions:**

The structured and rigorous development approach for this skin cancer risk reduction and brief behavioral intervention e-training for MTs begins to fill a gap in skin cancer risk reduction research. Iterative usability testing of our asynchronous Web-based training resulted in positive participant response. Our e-training approach offers greater learner accessibility, increased convenience, and greater scalability than the few existing programs and has the potential to reach many MTs nationally.

**International Registered Report Identifier (IRRID):**

DERR1-10.2196/13480

## Introduction

### Background

Skin cancer, the most common cancer in the United States, constitutes a serious public health burden [1-4]. Skin cancer may be deadly or disfiguring. The most serious skin cancer, melanoma, resulted in an estimated 9000 deaths in 2018 [4]. Skin cancer treatment costs approximated US $8.1 billion in 2011 [1-3]. Most skin cancers can be prevented by simple behaviors to protect the skin from ultraviolet radiation (UVR), such as staying in shade, wearing sun protective clothing, applying sunscreen, and avoiding indoor tanning [4,5]. Early detection of skin cancer greatly decreases its potential morbidity, mortality, and cost [6-8]. The probability of early skin cancer detection increases with full body visual skin assessment (VSA) [9]. Despite these effective prevention and early detection strategies, over 5 million skin cancer cases are diagnosed or treated annually [10]. Thus, decreasing the burden of skin cancer depends on concerted and innovative public health efforts that extend beyond the conventional biomedical practitioners to complementary and integrative health care practitioners. These efforts also involve other community-wide sectors and could incorporate electronic learning (e-learning) technology to allow widespread and easy dissemination of knowledge.

In 2014, the Surgeon General issued a *Call to Action to Prevent Skin Cancer*, endorsing comprehensive community-wide efforts to prevent skin cancer by diverse partners and sectors, including business, health care, and education [5]. Massage therapists (MTs) are community members typically practicing outside of conventional health care settings, yet are professionals involved in promoting health and wellness. Despite their interface with health and wellness, MTs have been overlooked as a community-based resource to (1) help promote skin cancer risk reduction and (2) reinforce consumer-targeted public heath skin cancer awareness messages.

MTs are uniquely positioned to promote skin cancer risk reduction through *eyes on the skin* observation and client-centered communication. During a typical full body massage, the client is unclothed under a drape. MTs systematically undrape and view each anatomical area, allowing the opportunity to visualize skin cancer risk factors such as sunburn, tanning lines, high mole counts, or suspicious lesions. Clients typically see their MTs more often and for longer durations than their primary care provider and are more likely to discuss health promotion [11-13], thereby providing greater opportunities for successful client-centered communication and encouragement of effective skin cancer risk reduction behaviors such as reducing UVR exposure [14].

In our prior work with MTs, we conducted in-person and Web-based tobacco cessation brief behavioral intervention (BBI) training for nonbiomedical health care practitioners (including MTs) in private practice contexts [15,16]. This electronic training (e-training) significantly increased practitioners’ use of client-centered communication, BBI, and referral skills in the form of offering clients a *helping conversation*. The helping conversation is a BBI that emphasizes active listening skills and motivational communication strategies to encourage and support clients’ healthy behavior change [15].

Skin cancer education and training for MTs has been inconsistent and not rigorously evaluated. Although many MTs receive some skin cancer education, the format, content, duration, source, and depth of this education varies [17]. The few skin cancer–focused in-person workshops and 1 Web-based course available to MTs [18] have not been systematically evaluated and, to our knowledge, do not include training for VSA, client risk assessment, client-centered communication, BBI, and referral skills [17].

### Objective

There is a need to develop more comprehensive, accessible skin cancer risk reduction training for MTs. Here, we describe the development of the Massage Therapists Skin Health Awareness, Referral, and Education (MTsSHARE) protocol, including the development of e-training technology, simulated client interactions, online data collection instruments, and in-person assessment of MTs’ application of their training. We will describe procedures for usability and feasibility testing of the training.

## Methods

### Phase 1 (Complete): Adapting Existing Programs and Development of Training and Assessment Technology

#### Conceptual Framework

Social cognitive theory (SCT) guided the overall study. Individuals learn and maintain new behaviors in a social context through reciprocal interaction of person, environment, and behavior. In total, 4 SCT constructs guided the overall training: (1) reciprocal determinism, or the dynamic and reciprocal interaction of MTs, their external social context, and behavioral responses to the training; (2) behavioral capability to have a helping conversation; (3) observational learning from e-training vignettes; and (4) self-efficacy, affecting behavior choices, efforts to overcome barriers to behaviors, and mastery of the behaviors [19]. According to SCT, observations of a behavior, in this case conversing with massage clients about skin health, influence observers’ perceived ability to perform the behavior (self-efficacy) and their perceived expected outcomes of the behavior, including strategies for effective performance.

To frame the BBI, we used the 4 steps of a helping conversation (awareness, understanding, helping, and relating), client-centered communication skills, client education and referral skills, and strategies for practice system involvement developed in our prior work [15]. The helping conversation framework emphasizes a brief motivational, client-centered approach that allows a range of MT behaviors in response to the situational context (eg, new, returning, or long-term client; massage routine; practice workflow) and the client’s readiness to change behavior—an approach more acceptable to MTs than proscriptive approaches to BBI used often in conventional health care contexts [15]. This framework is also easily adaptable for e-learning dissemination.

#### Formative Data Collection

To initiate training development, we conducted 5 key informant telephone interviews with subject matter experts (SMEs) who were licensed MTs in Arizona and had current or previous experience in MT education or online training. The interview responses illuminated strategies to engage MTs, assets to include in the training, and approaches for discussing health issues within MT practice. Specifically, the informants stressed the importance of considering the scope of practice throughout the training development (*don’t diagnose*) and the role MTs play in the health of their clients. They also helped establish the desired level of information throughout the training, for example, suggesting the inclusion of more detailed information regarding skin anatomy and the effects of UVR on the skin.

We then conducted 1 focus group with 5 additional locally practicing licensed MTs. The focus groups reviewed the themes that emerged during key informant interviews and generated data to further support the training. The key results highlighted the importance of the following:

- discussion of skin cancer risk reduction during appointments and why this activity is within the scope of practice;
- myriad ways to begin a conversation about skin cancer risk reduction with clients, including personal experience and nonjudgmental comments and questions;
- major barriers to conversing with clients about skin cancer risk reduction, such as lack of confidence and knowledge about skin cancer, and how to address these barriers;
- recommendations for how to teach MTs to have conversations with their clients about skin cancer risk reduction.

The informants focused on MTs’ ethical responsibility to share important health-related information with their clients, ask permission to chart any new or changing lesions noticed, and provide a list of local dermatologists for referral purposes.

#### E-Learning Module Development

Guided by our conceptual framework and formative data collection, we adapted content from 2 existing Web-based training programs: (1) a multimedia skin cancer risk reduction academic course, currently tailored for university students in the health sciences [20] and (2) MT client-centered communication and referral skills modules used in a BBI training for tobacco cessation [16].

We adapted skin cancer risk reduction content from the university’s academic course and the *Surgeon General’s Call to Action to Prevent Skin Cancer* [5] to include skin cancer risk factors, sun safety, VSA, and skin lesion assessment. We endeavored to provide MTs with a refresher of the information some may have received during their professional training, while focusing on content for the expected MT-client interaction and helping conversation. We adapted client-centered communication and referral skills content from previous studies that trained MTs to offer their clients helping conversations and referrals addressing tobacco cessation [15,16]. The 4 steps of a helping conversation as applied to skin cancer, awareness, understanding, helping, and relating, are described in Table 1.

Module development included (1) creating overall competencies and module-specific learning objectives, (2) reviewing existing curricula for structure, (3) adapting existing curricula resources or creating new multimedia content for e-learning, and (4) reviewing draft modules by SME, revising as needed. The e-training was asynchronous, interactive, and less than 2 hours in length, including accessing the modules via a Web-based learning management system, viewing and completing the modules, and completing study assessments. We chose Articulate Storyline for our e-learning course authoring software. Articulate Storyline provides the ability to create responsive modules that integrate audio, video, quiz, and activity components, allowing for a streamlined development and user interface experience.

The final training is based on 22 core competencies (see Table 1, column 2), adapted from the learning objectives of previous helping conversation-oriented Web-based training modules [15], which integrate the skin cancer content across the 4 steps of a helping conversation (see Table 1, column 1). The training comprises 6 modules (1) introduction, (2) awareness, (3) understanding, (4) helping, (5) relating, and (6) closing; each module contains photo and video media produced specifically for this project, as well as interactive activities that serve as *knowledge checks* focusing on specific content and skills.

SMEs in MT education, skin cancer, BBI training, online learning, public health, and information technology critiqued the content using an iterative process of review and structured/open-ended feedback proven successful in prior training projects [15]. The massage therapy SMEs were local and national opinion leaders, respected practitioners, and educators. Multimedia Appendix 1 contains screenshots from the Understanding module of the e-training.

**Table 1:** Massage Therapists Skin Health Awareness, Referral, and Education electronic training modules and competencies.

Helping conversation step and module name	Competencies	Content examples
*Awareness* (asking about/awareness of skin cancer risk/risk behaviors and opportunities to help)	Describe benefit of MTs as partners in skin cancer risk reduction; begin a helping conversation in a nonconfrontational and supportive way	Multimedia Appendix 1: Opportunity to help
*Understanding* (assessing readiness to change behaviors to reduce skin cancer risk and/or seek medical evaluation of a suspicious skin lesion and seeking understanding of the client’s motivations for/against behavior change)	Apply active listening skills: open-ended questions, clarifying questions, reflective questions/statements; use positive communication skills: express empathy, avoid problem solving, avoid lecturing, avoid arguing; assess and acknowledge major barriers to skin cancer risk reduction; elicit motivators that inspire risk-reducing behaviors; reinforce motivators that inspire risk-reducing behaviors; assess and acknowledge manageable risks for skin cancer; assess and acknowledge the client's willingness to take action; assess suspicious skin lesions; set realistic goals for the outcome of helping conversations	Multimedia Appendix 2: Understanding module screenshots
*Helping* (offering information about skin cancer risk reduction and referrals for medical evaluation)	Recognize how to offer support and encouragement based on the client’s risk profile and willingness to take action; identify different types of referral resources for professional help with skin health; provide information about professional skin health services; explain how the skills learned in this training can be applied in different situations	Multimedia Appendix 3: Returning client with suspicious lesion
*Relating* (arranging client follow-up on skin cancer risk reduction behaviors and referrals and offering ongoing encouragement for behavior change)	Seek permission to follow-up in a respectful manner; facilitate probability of follow-up by finishing the helping conversation on a positive note	Multimedia Appendix 4: Relating

#### Media Asset Development

We developed 5 brief MT-client interaction scenario example videos (average time of 30 seconds), using procedures from our previous consumer studies [21]. Specifically, we asked our SMEs in MT education to (1) review training content on helping conversation goals and skills, (2) reach consensus on the purpose of the video, and (3) review video scripts and storyboards. We recruited a convenience sample of consumers to read scripts and act as massage clients; our MT consultant acted in the MT role during video recording (see Multimedia Appendices 2 and 3 for example scenarios). We also recorded 5 testimonial videos (average time of 1 min) wherein the MTs described their experiences with providing skin cancer risk reduction information in their practices (see Multimedia Appendix 4 for example testimonials). We used the scenario and testimonial videos to enhance and reinforce topics discussed throughout the training by embedding the videos into the Web-based modules.

#### Development of Simulated Decision-Making Cases

**Figure 1:**
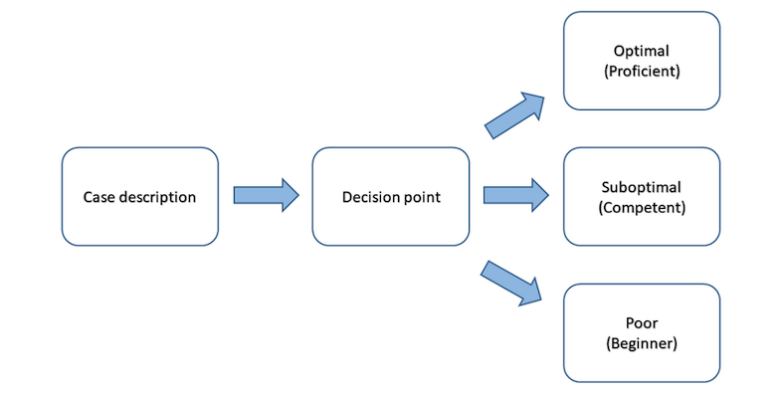
Decision path template.

**Figure 2:**
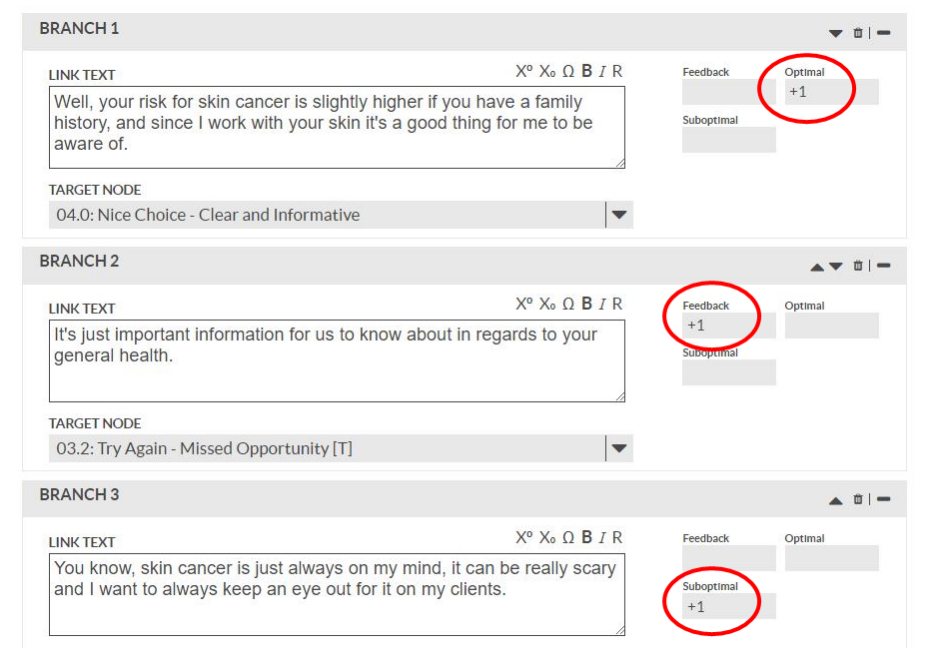
Screenshot of decision path tags.

Using procedures and cases from our previous BBI research [16], we developed electronic simulations of case-based practice of communication skills and application of skin cancer risk reduction knowledge using the Kynectiv DecisionSim [22] platform. Each case comprises skin cancer–focused scenarios that simulate MT decision making during a helping conversation. Furthermore, 3 MT-client interaction decision paths *optimal*, *feedback required*, and *suboptimal* are accompanied by a rubric for scoring each decision (see Figure 1). The rubric was informed by the competencies for the training modules (Table 1). Each response option is associated with a tag (in the back-end database) for each decision path (see Figure 2). To successfully complete the training, MTs must meet a minimum level of competence—selection of a response path that is within a specified range of the *ideal* interaction path (eg, appropriate MT response during a helping conversation when a client is open to a discussion about skin cancer risk reduction behavior vs a different MT response if the client is resistant to discussion). We developed 5 case simulations for participants to complete following the 6 training modules.

#### Development of Learning Management System

The learning management platform chosen to host the training modules was Desire2Learn (D2L; Desire2Learn Inc), which could seamlessly link to the case simulations. D2L allowed us to design the course components and navigation to facilitate engagement and ease of use. We designed course content to be accessed sequentially, requiring the completion of a module before accessing the next one; this ensures that participants complete the modules in the intended order, but at their own pace. The course home page features a resources section with downloadable PDFs, intended for both MT and client use. These provide skin cancer–related information, as well as tips for offering helping conversations.

#### Development and Adaptation of E-Learning Data Collection Methods and Assessments

The study personnel collected and managed study data using the Research Electronic Data Capture (REDCap) electronic data capture tool hosted at the University of Arizona [23]. REDCap is a secure, Web-based application designed to support data capture for research studies, which provides (1) an intuitive interface for validated data entry, (2) audit trails for tracking data manipulation and export procedures, (3) automated export procedures for seamless data downloads to common statistical packages, and (4) automatic triggering of surveys and email correspondence.

We selected and modified our training assessments from the literature and those used in previous research. Participant assessments are timepoint-specific versions of 2 surveys: (1) baseline survey to assess participants’ sociodemographic and practice-related data, as well as skin cancer risk reduction knowledge and (2) a case-based skin lesion image assessment that allowed participants the opportunity to view images of skin lesions and determine whether they were suspicious and prompted referral to a physician, or nonsuspicious. The baseline survey was modified into a posttest without sociodemographic data to assess knowledge and practice-related behaviors immediately posttraining and at 3 and 6 months.

We also developed a 5-item client survey to be advertised in participating MT offices and lobbies, inviting all clients of participating MTs to anonymously share whether their MT engaged in skin cancer risk reduction conversations during their massage visit. To further validate the MTs’ application of the training, we asked super clients to conduct an immediate postmassage assessment. A super client is a study participant who participates in the study by receiving a massage and assesses MT’s use of helping conversations pertaining to skin cancer risk reduction. This in-person observational assessment was adapted from the concept of unannounced standardized patients commonly used in medical and clinical education [24].

#### Iterative Usability Testing

The University’s Institutional Review Board approved all human subject procedures for iterative user testing of the MTsSHARE e-training. To assess the usability of the training modules, assessments, and procedures before implementing a larger quasi-experimental longitudinal study with Arizona MTs, we enrolled a convenience sample of 20 licensed MTs from throughout the United States (except Arizona). We used the predetermined feasibility study eligibility criteria to determine MT eligibility (see below), enrolling eligible MTs in 4 waves of 5. Following all training and assessment components, participants completed a 27-item usability survey adapted from the feedback form used by SMEs during module development. The usability survey questions focused on course content (the content of the modules is at the appropriate level for MTs), accessibility (it is easy to access the helping conversation simulations), and relevance (the content in the modules is relevant to my (MT’s) practice). We scored all items on a 5-point scale from strongly disagree to strongly agree. After each wave of 5, we analyzed responses and made appropriate changes to study protocol, assessment, and training components as necessary. We coded open-ended responses for major categories. The massage therapy SMEs were local and national opinion leaders, respected practitioners, and educators categories using established methods for qualitative content analysis [25]. The usability assessment was conducted from March 3, 2018 to July 31, 2018.

The overall mean scores for usability slightly improved with each iteration, increasing from a low score of 3.5 to a 5 (moderately agree to strongly agree), with an overall usability mean score of 4.96. However, the key findings from usability testing were the appropriateness of simple, seamless technology, the progression and relevance of the information presented, suggestions for additional content and general instruction, and the utility of including interactive assessments and client simulation exercises. Making changes after each wave resulted in the progressive improvement of the modules. The final version of the e-training tested well for usability and satisfaction.

### Phase 2 (In Progress)

#### Feasibility Study Design

**Figure 3:**
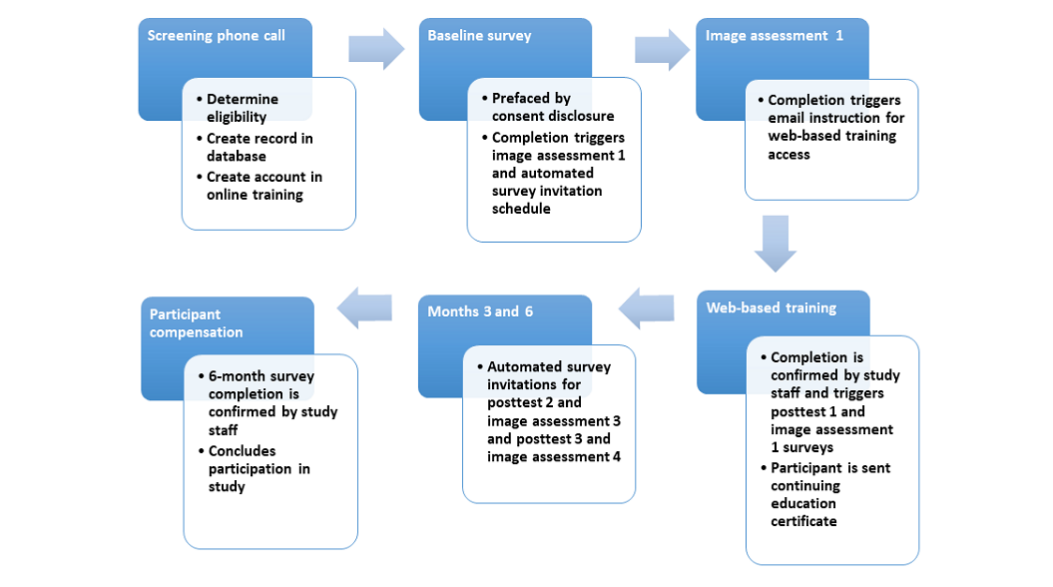
Feasibility study protocol.

The feasibility study is a single cohort design (see Figure 3) with participant assessments at 4 time points: (1) immediately upon study enrollment (baseline survey and image assessment 1), (2) posttest 1 occurring immediately after training completion and image assessment, (3) posttest 2 occurring 3 months after training completion and image assessment, and (4) posttest 3 occurring 6 months after training completion and image assessment. After completing the e-training, participants receive an electronic gift card and a certificate for 1 hour of continuing education (CE), approved by the National Certification Board for Therapeutic Massage and Bodywork.

A subset of 20 Tucson-based MTs will receive a visit from a trained super client at least 3 months after completing the e-training.

The phase 2 of the study, participant enrollment and data collection, is ongoing. All survey invitations are delivered via automated email from the REDCap system, triggered by items completed in an administrative survey by study staff or timepoints based on completion of the e-training.

#### Participant Recruitment, Eligibility, and Enrollment

Our goal for enrollment is 80 MTs practicing in the state of Arizona. We based our sample size estimation on the published literature on skin cancer training for medical students [26] and our own research on tobacco cessation BBI with licensed MTs. Given the potential for a high attrition rate experienced in Web-based trainings [27], we concluded that a sample of 80 participants is sufficiently powered and allows for possible attrition. The sample size analyses were conducted using PASS (V.12). This sample size is large enough to reasonably estimate, in conjunction with sensitivity analysis, relevant variance components, recruitment, and dropout rates for use in a future definitive trial [28].

To be eligible, MTs must be aged at least 21 years, be a licensed MT in the state of Arizona, have practiced for at least 3 years, provide mainly full body massages, have access to computer with broadband internet connection and audio, and agree to forego continuing education on skin cancer for the duration of study participation. Excluded are MTs who have received continuing education on skin cancer, sun safety or client communication skills training in the past 2 years, and those who perform only partial body massages.

The recruitment began with Arizona-based MTs who initially responded to the recruitment efforts for usability testing. We then contacted state and national MT organizations, as well as Arizona-based leaders in the massage therapy business, previously known to study staff, to share recruitment materials via social media accounts.

Interested MTs email or call the designated initial contact study staff member, who enters the MTs’ information into a shared recruitment database in REDCap. The study staff then schedule and conduct a screening phone call. During this call, if the MT assents verbally to study participation, study staff enroll the MT into the REDCap system, which immediately sends the baseline survey link containing the electronic consent disclosure.

#### Participant Training and Follow-Up (In Progress)

We designed the REDCap database to send an automated notification of completion of image assessment 1 to study staff; this instructs study staff to send the e-training login instructions to the participant via email. The participants have 2 weeks to complete the 6 Web-based training modules and 5 DecisionSim cases. The study personnel check daily for training completion, emailing the certificate for 1 hour of continuing education, and logging the date of completion in the administrative survey. Completion of the training triggers posttest 1 and image assessment 2. When a participant completes the training, study personnel distribute client survey flyers to the MT in person, electronically or via US mail.

#### Super Client In-Person Observational Assessment

Super clients will visit Tucson-based participating MTs for an average 60-min full body massage. Super clients will receive a simple henna tattoo by study personnel, imitating a suspicious lesion, placed on their foot or ankle region before their first massage; this tattoo will be identical on each super client and will serve as a standardizing feature. Following their massage, the super client will complete a brief electronic survey about their massage experience, focusing on whether their MT engaged in a conversation about skin cancer risk reduction and whether they mentioned the suspicious lesion (henna tattoo).

To date, we have enrolled and trained 5 super clients (4+1 alternate), who will each visit 5 MTs, for a total of 20 MTs visited. We selected a convenience sample of super clients to represent a variety of demographic characteristics (age, gender, phenotype, health history, and sun protective behaviors).

#### Data Analysis

Feasibility outcomes, including recruitment and dropout, training completion, overall client feedback, and MT satisfaction, will be described using frequencies and percentages and 95% CIs.

The longitudinal measures will use appropriate mixed models (linear for continuous outcomes and generalized linear with a logistic link for binary) using time categorically to protect against model misspecification. Comparisons of baseline with 3- and 6-month measures will be carried out using contrasts within these models. The mixed-models are robust to missing outcome data (including dropout) and model misspecification [29,30].

Sensitivity/specificity across 4 timepoints will be compared for image assessment scores. For each timepoint we will assess the following parameters for image assessment: sensitivity, specificity, the likelihood ratio for a positive result, and the likelihood ratio for a negative test result. We will evaluate separate bivariate logistic regression models for each set of image assessments to determine the odds ratio in predicting the correct image. We will evaluate separate models including the scores for each timepoint to determine the areas under the receiver operating characteristic (ROC) curves for image assessments. The area under the ROC curve measures the probability of correctly identifying a true negative (not suspicious) or true positive (suspicious) image.

Client survey data and super client data will be analyzed with descriptive statistics. We will correlate scores from the super client assessment with the DecisionSim scores to further validate MTs’ application of helping conversation skills learned in the training. We will conduct an optional debriefing webinar for the 80 MTs in the third year to gain further information about their experience with the curriculum.

Mixed-effects linear regression models for longitudinal data will be ﬁtted to evaluate intervention outcomes adjusted for participant characteristics, for example, age, gender, years in practice, geographical area, and client workload. The mean differences in each of the primary outcomes will be evaluated in separate models, including the covariates as fixed effects and subjects as random effects. We may also consider geographical area as random effect (urban vs rural). In this case, geographical area and subjects will be fitted as random effects to account for the correlation within geographical area and serial intrasubject correlations. Predictor variables with multiple categories will be entered as indicator variables. For dichotomized intervention effects, we will use mixed-effects logistic regression models.

## Results

For Phase 2, we have screened 77 MTs who have expressed interest in participating. Of those, 14 were either not interested or not eligible (either lacked time to participate or did not see an average of 10 clients per week) and 15 did not follow up after contact attempts were made. We enrolled and consented the remaining 48 MTs. At the time of the paper submission, 11 enrolled MTs had dropped from the study, owing to lack of time to participate. Of the 37 MTs still enrolled, 32 have completed the training, with the remaining 5 having begun but not yet completed the training. We will close recruitment in August 2019.

## Discussion

### Principal Findings

The current prevention and early detection strategies have not had a significant impact on reducing the public health burden of skin cancer [5]. We used a rigorous strategy to enlist MTs as partners in skin cancer prevention and detection, developing innovative e-training and assessment protocols. The American Massage Therapy Association estimates that there are 335,000 to 385,000 licensed or certified MTs in the United States, who see about 39.1 million clients annually [31]. Thus, MTs are a largely untapped resource for reducing skin cancer risk. A search of MT training on PubMed and Google Scholar revealed that most articles focus on massage therapy as an intervention and the health outcomes of massage. Few scholarly articles addressed training MTs for a specific skill following their primary professional education [15,16]. Muramoto et al [16] were the first to successfully develop and implement e-training (BBI training and competency evaluation) of complementary and alternative medicine providers (including MTs) for screening clients for tobacco use and encouraging tobacco cessation. These authors also were the first to develop e-training for these specialized providers.

The structured and rigorous development approach for this skin cancer risk reduction and BBI e-training for MTs begins to fill a gap in skin cancer risk reduction research. We surveyed 100 MTs in an elecronic, national survey where we asked for the MTs’ perceptions of conversations with clients related to skin cancer prevention, as well as detection [32]. The 2 published studies have targeted skin cancer risk reduction in convenience samples of MTs who were attending national MT conferences. One study surveyed 262 MTs to assess their comfort level regarding potential assessment of suspicious skin lesions [17]. The other study reported findings from a face-to-face, 4-hour education session that provided information only to 114 MTs [33]. No previous studies have addressed how MTs could integrate this information into the context of a client visit via client education, or communication skills, such as a BBI (helping conversation) to encourage skin cancer risk reduction. Our e-training approach offers greater learner accessibility, increased convenience, and greater scalability [34]. Thus, the e-training format has the potential to reach many more MTs, nationally.

We found few other e-learning opportunities pertaining to skin cancer, most of which targeted conventional health care providers with a goal of increasing competencies in diagnostic knowledge and skills competency [35-37]. These ranged from several Web-based modules to video training delivered by electronic links [37]. In these studies, providers had a positive impression of the Web-based curriculum, and in one case, increased the likelihood of discussion with patients about skin cancer. The accessibility, effectiveness, and popularity of the curriculum indicated potential for implementation in the primary care setting. Our e-training is designed to be brief, yet engaging, informative, and integrated into the context of a typical client visit to an MT. MTs can access the training when convenient and move from one module to the next at their own pace, both of which are important for learner control and engagement [38].

Previous training targeting MTs did not appear to be pilot tested or assessed for usability. Our use of formative and summative evaluations along with predesignated *stopping rules* (ie, 4 iterations) represented the ideal conceptualization of usability [39]. The ease of navigation of the training modules and available resources made this training appealing to the participating MTs. The training incorporated highly interactive, scenario-based, simulated helping conversations focused on skin cancer risk reduction, and the simulations provided participants with opportunity to interact with the training, apply knowledge gained, and practice skills learned, reflecting the SCT theory. These features also are important to enhancing e-learning [34].

### Barriers and Opportunities

The preliminary results reveal anticipated difficulties with recruitment and retention within the MT population. No previously published studies of MTs as participants have addressed recruitment challenges. For the in-progress feasibility study, barriers to enrollment have related to practice-related eligibility conditions, such as number of years in practice and number of clients seen per week. The primary barrier to retention following enrollment has been a self-professed lack of time to participate. It is encouraging that, of the eligible and enrolled MTs, 67% (32/48) have completed the training and progressed to follow-up assessments. Offering incentives in the form of monetary compensation, as well as continuing education credit, has been a useful approach to address both recruitment and retention.

This paper provides an overview of our systematic approach to developing rigorous e-training for MTs to enable them to be partners in skin cancer risk reduction. The phase 2 results will explicate the feasibility of the e-training approach for further efficacy testing.

## Appendix

Multimedia Appendix 1 Opportunity to help.

Multimedia Appendix 2 Understanding module screenshots.

Multimedia Appendix 3 Returning client with suspicious lesion.

Multimedia Appendix 4 Relating.

## Abbreviations

BBI: brief behavioral intervention
D2L: Desire2Learn
e-learning: electronic learning
e-training: electronic training
MT: massage therapist
MTsSHARE: Massage Therapists Skin Health Awareness, Referral, and Education
REDCap: Research Electronic Data Capture
ROC: receiver operating characteristic
SCT: social cognitive theory
SME: subject matter expert
UVR: ultraviolet radiation
VSA: visual skin assessment
