# Supplementary material for: Web-Based Skin Cancer Prevention Training for Massage Therapists: Protocol for the Massage Therapists Skin Health Awareness, Referral, and Education Study
Source: JMIR Res Protoc. 2019 May 15;8(5):e13480. doi: 10.2196/13480 (PMC6540726; doi:10.2196/13480)
Supplement: Multimedia Appendix 2 [file resprot_v8i5e13480_app2.pdf]

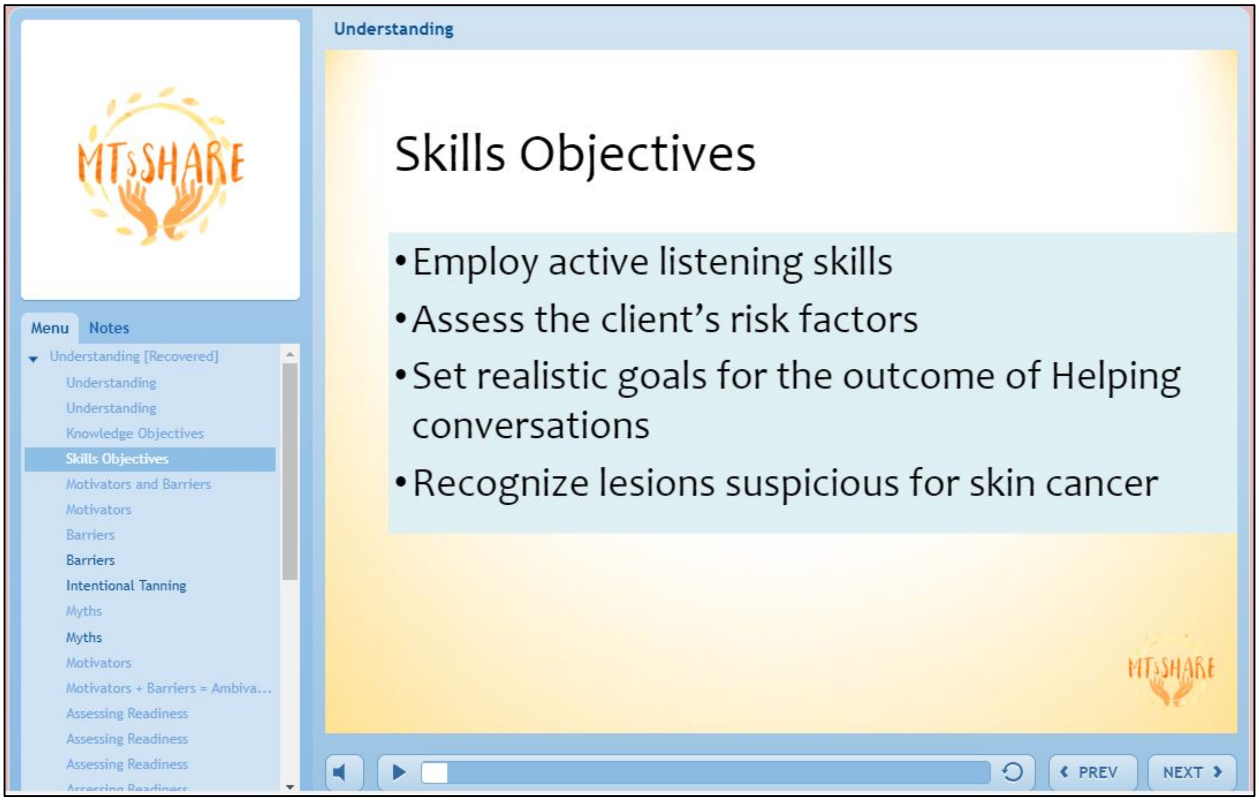
Understanding
Skills Objectives
• Employ active listening skills
• Assess the client's risk factors
• Set realistic goals for the outcome of Helping conversations
• Recognize lesions suspicious for skin cancer
MTsSHARE
Menu
Notes
Understanding [Recovered]
Understanding
Understanding
Knowledge Objectives
Skills Objectives
Motivators and Barriers
Motivators
Barriers
Barriers
Intentional Tanning
Myths
Myths
Motivators
Motivators + Barriers = Ambiva...
Assessing Readiness
Assessing Readiness
Assessing Readiness
PREV
NEXT

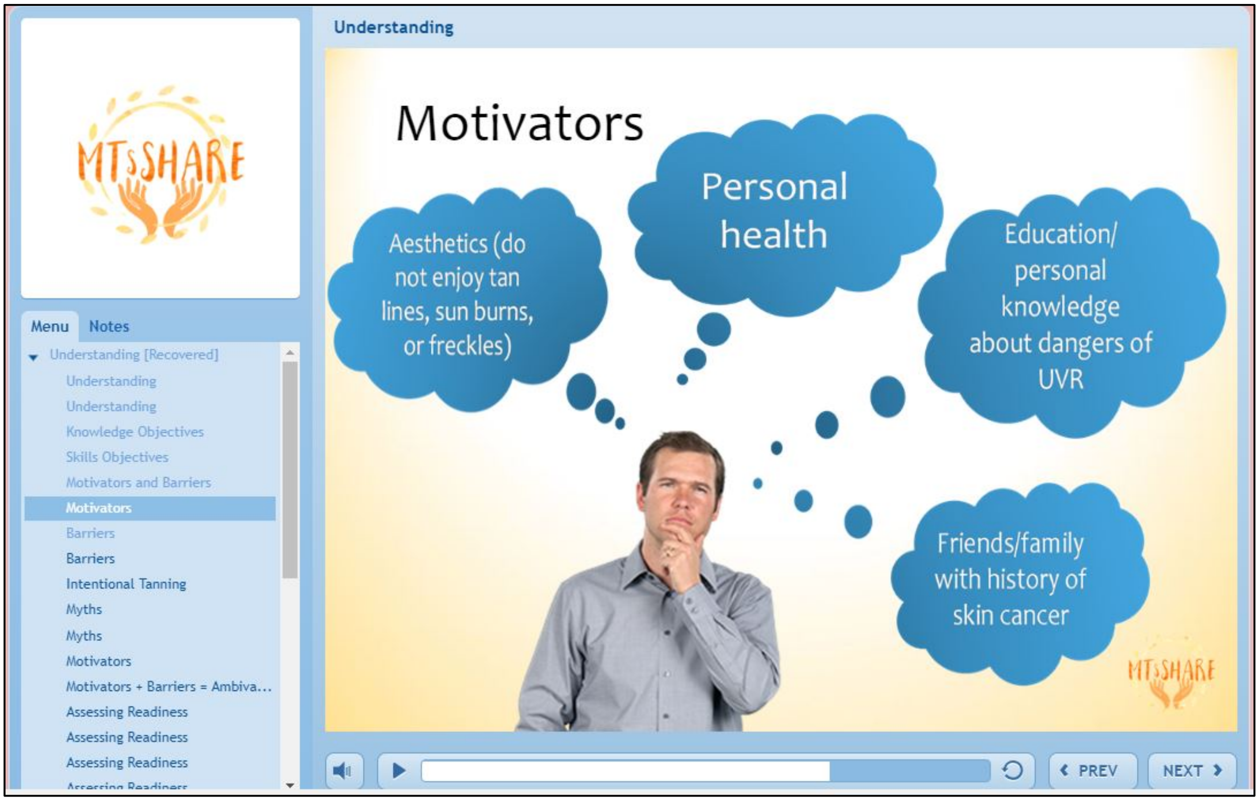
Understanding
Motivators
Aesthetics (do not enjoy tan lines, sun burns, or freckles)
Personal health
Education/ personal knowledge about dangers of UVR
Friends/family with history of skin cancer
MTsSHARE
Menu
Notes
Understanding [Recovered]
Understanding
Understanding
Knowledge Objectives
Skills Objectives
Motivators and Barriers
Motivators
Barriers
Barriers
Intentional Tanning
Myths
Myths
Motivators
Motivators + Barriers = Ambiva...
Assessing Readiness
Assessing Readiness
Assessing Readiness
PREV
NEXT

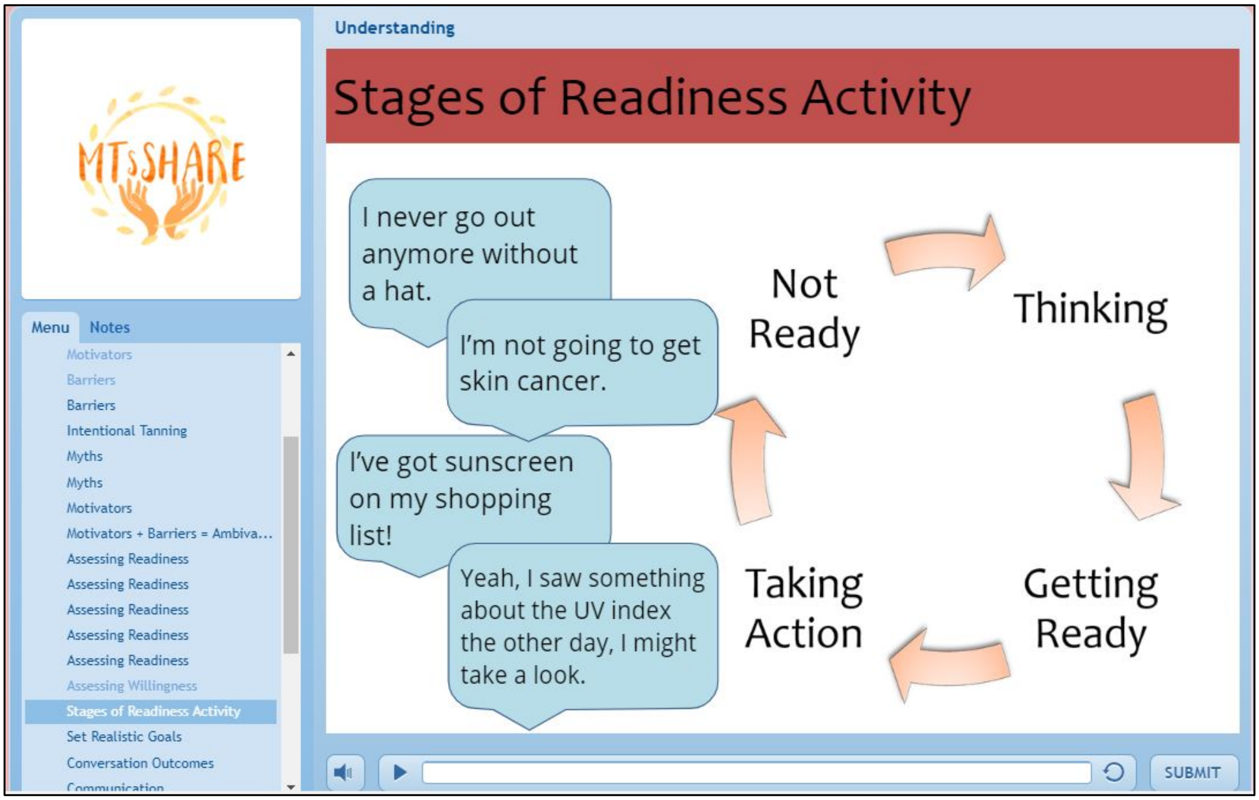
Understanding
Stages of Readiness Activity
I never go out anymore without a hat.
I'm not going to get skin cancer.
I've got sunscreen on my shopping list!
Yeah, I saw something about the UV index the other day, I might take a look.
Not Ready
Thinking
Getting Ready
Taking Action
MTsSHARE
Menu
Notes
Motivators
Barriers
Barriers
Intentional Tanning
Myths
Myths
Motivators
Motivators + Barriers = Ambiva...
Assessing Readiness
Assessing Readiness
Assessing Readiness
Assessing Readiness
Assessing Readiness
Assessing Willingness
Stages of Readiness Activity
Set Realistic Goals
Conversation Outcomes
SUBMIT

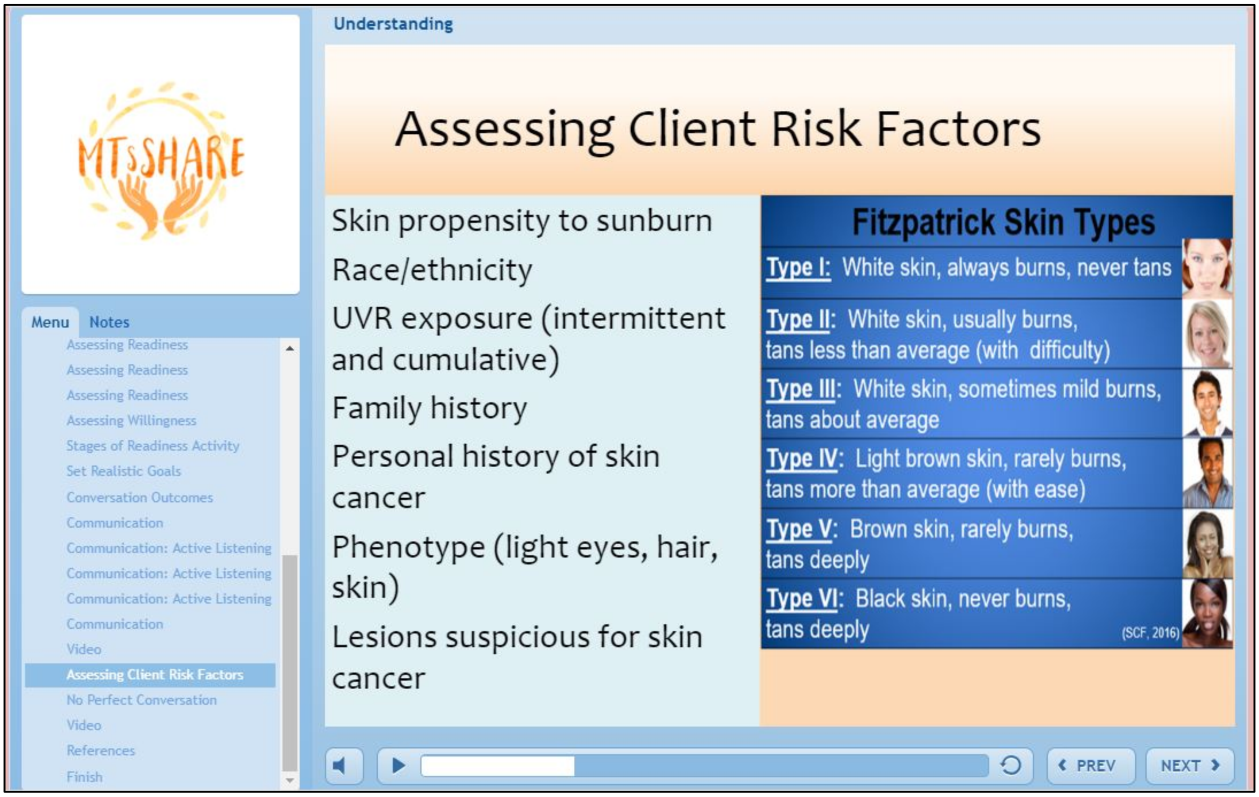
Understanding
Assessing Client Risk Factors
Skin propensity to sunburn
Race/ethnicity
UVR exposure (intermittent and cumulative)
Family history
Personal history of skin cancer
Phenotype (light eyes, hair, skin)
Lesions suspicious for skin cancer
Fitzpatrick Skin Types
Type I: White skin, always burns, never tans
Type II: White skin, usually burns, tans less than average (with difficulty)
Type III: White skin, sometimes mild burns, tans about average
Type IV: Light brown skin, rarely burns, tans more than average (with ease)
Type V: Brown skin, rarely burns, tans deeply
Type VI: Black skin, never burns, tans deeply
(SCF, 2016)
MTsSHARE
Menu
Notes
Assessing Readiness
Assessing Readiness
Assessing Readiness
Assessing Willingness
Stages of Readiness Activity
Set Realistic Goals
Conversation Outcomes
Communication
Communication: Active Listening
Communication: Active Listening
Communication: Active Listening
Communication
Video
Assessing Client Risk Factors
No Perfect Conversation
Video
References
Finish
PREV
NEXT
